# Scenario Generation for Autonomous Vehicles with Deep-Learning-Based Heterogeneous Driver Models: Implementation and Verification

**DOI:** 10.3390/s23094570

**Published:** 2023-05-08

**Authors:** Li Gao, Rui Zhou, Kai Zhang

**Affiliations:** 1 Shenzhen International Graduate School, Tsinghua University, Shenzhen 518000, China; 2 Research Institute of Tsinghua, Pearl River Delta, Guangzhou 510530, China; 3 Institute of Systems Engineering, Macau University of Science and Technology, Macau 999078, China; 4 Waytous Inc., Shenzhen 518000, China

**Keywords:** scenario generation, heterogeneous driver model, deep learning, autonomous-driving testing

## Abstract

Virtual testing requires hazardous scenarios to effectively test autonomous vehicles (AVs). Existing studies have obtained rarer events by sampling methods in a fixed scenario space. In reality, heterogeneous drivers behave differently when facing the same situation. To generate more realistic and efficient scenarios, we propose a two-stage heterogeneous driver model to change the number of dangerous scenarios in the scenario space. We trained the driver model using the HighD dataset, and generated scenarios through simulation. Simulations were conducted in 20 experimental groups with heterogeneous driver models and 5 control groups with the original driver model. The results show that, by adjusting the number and position of aggressive drivers, the percentage of dangerous scenarios was significantly higher compared to that of models not accounting for driver heterogeneity. To further verify the effectiveness of our method, we evaluated two driving strategies: car-following and cut-in scenarios. The results verify the effectiveness of our approach. Cumulatively, the results indicate that our approach could accelerate the testing of AVs.

## 1. Introduction

As challenging driving scenarios rarely occur in reality, traditional on-road testing is not worth the time and money [1]. According to the 2021 annual disengagement reports of the Department of Motor Vehicles (DMV), the autonomous driving road test mileage reached 4.1 million miles in 2021, surpassing the previous reporting cycle by 2 million miles. However, the highest miles per intervention (MPI) exceeded 50,000 miles. Although specific challenging scenarios can be created manually, some conditions, such as extreme weather, are difficult to modify. Scenario-based simulation testing is a reliable solution to the problem of road testing [2,3,4,5].

Although low-cost and efficient scenario-based virtual testing has attracted more attention, testing all scenarios is also a waste of computing resources. Accelerated evaluation focuses on finding representative scenarios such as small probability events that may violate autonomous vehicles’ (AVs) safety requirements [6,7]. For example, Zhao et al. used importance sampling to effectively sample rare events, achieving the same test results, but with fewer scenarios [8]. Huang et al. proposed a piecewise model as a more flexible structure to capture the tails of the data more accurately [9]. Furthermore, Althoff et al. combined reachability analysis and optimization techniques to reduce the size of the solution space for autonomous vehicles [10]. The above three methods use empirical distributions, but it is also possible to focus on only part of the particular space. Sun et al. summarized three types of methods for finding partially unsafe scenarios by delineating boundaries [11]: finding high-risk scenarios [12], boundary scenarios [13], and collision scenarios [14].

However, the sampling space for those acceleration approaches is fixed because all environmental vehicles utilize the same model and cannot be adjusted. Aggressive drivers are more likely to behave in a manner that endangers others [15,16,17]. Therefore, we could consider environmental vehicles variables, and by controlling these variables, we could adjust the proportion of hazardous situations in the scenario space. Ge et al. tried to describe driving behavior with utility functions [18]. Different drivers’ driving strategies can be represented by different utility functions. Modifying the driving strategies of surrounding vehicles (SVs) can result in more challenging events for the AV. A utility function defines the selection of a particular behavior, such as the likelihood of lane changing or the following distance [19]. A control model is also needed to calculate the speed of SVs.

**Figure 1:**
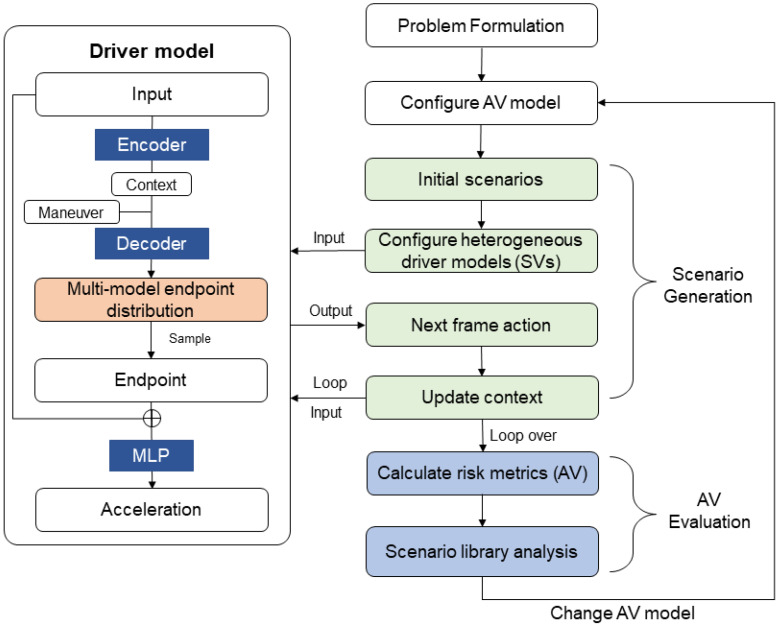
An overview of the work in this paper.

Designing a driver model that simulates human behavior for environmental vehicles is necessary to satisfy the uncertainty of human behavior. Imitation learning is a common approach, but it requires the manual definition of cost functions and is computationally expensive [20]. Aksjonov et al. used an artificial neural network (ANN) to predict human behavior and achieved good performance [21]. We could equate driver modeling to the trajectory prediction problem. Yang et al. used a Gaussian mixture model to identify the driving style, and proposed a personalized joint time series modeling method for trajectory prediction [22]. Such methods are deterministic predictions that cannot handle the multiple possibilities of human behavior. To explore uncertainty about future states, some methods predicted multiple possible paths. Zhao et al. estimated the endpoint candidates with high probability on the basis of the environmental context and generated trajectories [23]. Tian et al. proposed a joint learning architecture to incorporate the lane orientation, vehicle interaction, and driving intention in multi-modal vehicle trajectory forecasting [24]. The GAN-based method incorporates latent variables into network learning and optimizes the generated trajectories [25]. However, these stochastic prediction methods do not represent driver heterogeneity well. For scenario sampling, the prediction target is not the driver’s optimal trajectory [26,27], but the probability distribution of the next action. Deo et al. utilized the information of surrounding vehicles to predict multimodal trajectory distributions [28]. According to this knowledge, we propose heterogeneous driver models with integrated decision and control implemented with deep learning. Heterogeneity is reflected in separately training the models with different driver data types. Scenarios are generated through real-time interaction between SVs with our driver model and AVs. Taking five common initialization scenarios as examples, we changed the model style of the SVs to obtain more dangerous events. To demonstrate that our method works, we evaluated the two driving strategies with the generated scenarios. Compared with the above methods, our method could better accelerate evaluation. The overview of our work is depicted in Figure 1.

The contributions of this paper are as follows:

(1) Uncertainty in driver behavior is learned using deep-learning methods for the dynamic generation of stochastic scenarios.

(2) Driver heterogeneity is demonstrated to be able to generate more realistic and complex scenarios, and in some cases, increase the proportion of critical scenarios.

(3) Autonomous vehicles with scenarios generated by our method were tested, and the safety and efficiency of two driving strategies were evaluated.

The rest of this paper is structured as follows: Section 2 introduces our scenario generation method. Section 3 analyzes our experimental results, and Section 4 shows the conclusions.

## 2. Scenario Generation Method

This section describes the definition of our scenario generation problem and introduces the heterogeneous driver models for environmental vehicles.

### 2.1. Problem Description

The scene of time step t can be defined as $x_{t}=\left(d,v,a,rd\right)$, where *d* is the coordinate, *v* is velocity, *a* is acceleration, and rd is relative distance. Scenario $X=(x_{t},x_{t+1},...)$ is defined as a sequence of scenes. Initializing the scene sequence for each vehicle, we describe the scenario generation problem as sampling a new scene sequence through the interaction between objects. The driver model inputs historical scene information, $I=(x_{t-p},...,x_{t-1})$, and makes decisions first. *p* is the observation window. $P(m_{i}|I)$ is known for a given *I*, where m indicates lane-change and braking events. The decision results were sampled from this distribution. Moreover, a distribution for sampling acceleration needs to be found. Directly predicting the acceleration distribution’s next frame and sampling it leads to an unsmooth vehicle trajectory [29]. Therefore, we predicted the distribution of endpoints in the future observation time and sampled an endpoint from it as driver intent feature. The probability of a control event can be written as follows:

(1) $$P\left(a|I\right)=\sum P\left(a|Y\right)P_{\theta }\left(Y|m_{i},I\right)P\left(m_{i}|I\right)$$

where $P_{\theta }\left(Y|m_{i},I\right)$ denotes the probability of future endpoints. The parameter θ is obtained via model learning. The coordinate system must always have self-location at time *t*− 1 as its origin for the model to be valid at any location. If the original coordinate sequence is $\left(d_{t-p},...,d_{t-1}\right)$, the coordinate conversion calculation formula is defined as follows:

(2) $$d_{t-i}^{{}^{\prime }}=d_{t-i}-d_{t-1}$$

AV acceleration is calculated with a car-following control model. The other parameters of $x_{t}$ can be calculated via a, which denotes a generated scene. The above process is repeated until AV passes the test or a collision occurs.

### 2.2. Datasets

We trained the model using the public highD dataset [30] containing UAV data recorded on German highways, including the trajectory information of more than 110,500 vehicles sampled at 25 Hz. We down-sampled the trajectory data to 5 Hz to improve the training speed. Each track’s data contain coordinates, speed, acceleration, and the surrounding vehicle ID.

**Figure 2:**
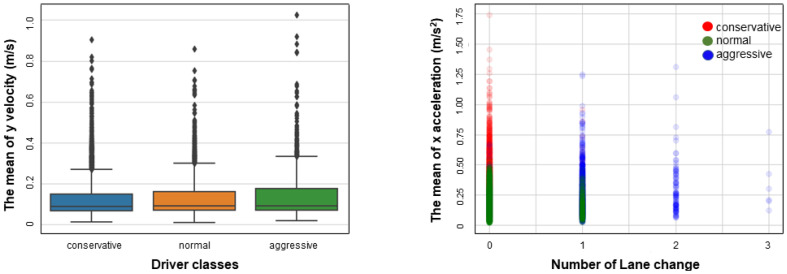
Feature visualization for the three driver classes.

To train the heterogeneous driver models, we had to classify the dataset. Drivers could be divided into three categories according to style: aggressive, normal, and conservative. We used the k-means algorithm to cluster all drivers into these categories on the basis of the mean, variance, and maximal values of velocity and acceleration. Figure 2 visualizes some of the features of each cluster. Aggressive drivers perform more lane changes and a wide range of longitudinal acceleration, while conservative drivers tend to maintain their lane and change speed smoothly. Our models learn their properties separately.

### 2.3. Heterogeneous Driver Modeling

Acceleration calculation in scenario generation should not be considered a simple regression task. Spatial navigation awareness drives a person to reach a predetermined area and plan a route [31]. Their actions change with the scene context and intentions at any time. On the basis of this observation, we propose a novel two-stage driver model that first estimates the maneuver probability and then generates endpoint areas on the basis of a sampled maneuver for planning actions.

**Figure 3:**
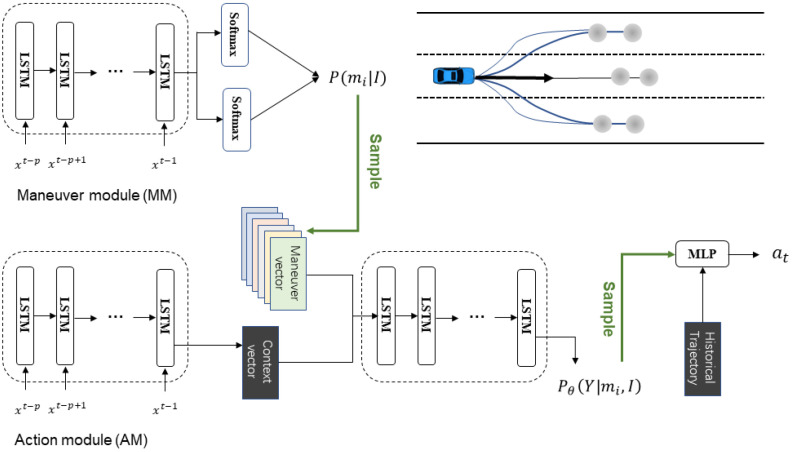
The structure of the proposed driver model.

Figure 3 shows an overview of our model, consisting of two components:

- Maneuver model (MM): estimates maneuver probabilities from the scene context.
- Action model (AM): Generates possible future terminal areas on the basis of selected maneuvers and then samples the endpoint from the terminal area as the intention feature. The endpoint and historical trajectory serve as input to generate the next action.

#### 2.3.1. Maneuver Module (MM)

We consider six maneuver classes. Lateral maneuvers are left-lane change, right-lane change, and maintaining the current lane. Lane changing takes about 6 s from start to finish. Therefore, the observation window was set to 3 s. The lane-changing state is defined as the lane ID change within the observation window. Longitudinal maneuvers are braking and normal driving. Braking is defined as the average speed of the next 3 s being less than 0.9 times the average speed of the historical 3 s [32]. MM consists of a long short-term memory (LSTM) encoder concatenated with two softmax functions. We sampled a maneuver from the conditional probability, $P(m_{i}|I)$, as the input of the action module.

#### 2.3.2. Action Module (AM)

According to the output of the MM, a maneuver is randomly sampled as the premise of the driver’s intention. Specifically, the ground truth is used during training. We need a sampling space that represents all scenes. Environmental information is encoded into context vectors. When decoding, the context vector is concatenated with the selected maneuver, and a five-dimensional vector representing the parameters of the Gaussian endpoint distribution is output. An endpoint is sampled in this distribution as the intention feature, and it is input to the MLP together with the historical trajectory information to generate the acceleration of the next frame, which can better reflect the randomness of behavior. We experimentally confirmed that the stochasticity of action was reflected well.

The AM consists of a classic LSTM encoder–decoder [33] and a multilayer perceptron (MLP) [34]. The encoder–decoder framework estimates the sampling space for the short-term endpoint region. An MLP is a fully connected class of a feedforward artificial neural network (ANN). The encoder is the same as that in MM. It can extract the displacement information and relative position to the surrounding eight vehicles.

#### 2.3.3. Model Training

During training, the objective function could minimize the following error. Because there are few lane-changing categories in the dataset, to reduce the impact of data imbalance, we chose to minimize the focal loss of the maneuver category, which adds a weight factor to the loss function to increase the weight of the minority category in the loss function [35], noted as follows:

(3) $$l_{focal}=-\alpha_{t}{\left(1-p_{t}\right)}^{\gamma }log\left(p_{t}\right).$$

For the 2D Gaussian distribution of the endpoint, we chose to minimize its negative log likelihood loss [36] as follows.

(4) $$l_{nll}=0.5*\left(log\left(max\left(var,eps\right)\right)+\frac{y-\hat{y}}{max(var,eps)}\right).$$

We used the mean squared error [36] for the sampled end point and next frame acceleration as follows.

(5) $$l_{mse}=\frac{1}{n}\sum {\left(Y_{i},\hat{Y_{i}}\right)}^{2}.$$

Therefore, the objective function was defined as follows.

(6) $$L=l_{nll}+l_{focal}+l_{mse}$$

We used LSTMs with 128 units and an MLP with 3 hidden layers. Heterogeneous models were obtained by training separately for each style. All models are trained using Adam with a learning rate of 0.001. The models were implemented using pyTorch. As a comparison, we also trained an original driver model without considering heterogeneity using the unclassified dataset.

Because accuracy is not a critical part of our method and does not affect the results of this paper, we later focused on the generated scenarios and did not compare the training results with those of other methods.

### 2.4. Implementation and Verification

We designed experiments to demonstrate the effect of heterogeneity on the number of challenging scenarios. To further verify the effectiveness of our scenario generation method, we evaluated the performance of two driving strategies by testing AV with our generated scenarios. This section introduces the autonomous driving model, driving strategies, and experimental scheme.

#### 2.4.1. Intelligent Driver Model

In this work, AVs were implemented using the intelligent driver model (IDM) [37], a car-following model with longitudinal control. It aims to calculate the desired speed and distance on the basis of the current vehicle speed and relative distance. The basic definition is as follows:

(7) $$\dot{a}=a_{max}\left[1-{\left(\frac{v}{\tilde{v}}\right)}^{\beta }-{\left(\frac{\tilde{s}}{s}\right)}^{2}\right]$$

where $a_{max}$ is the maximal acceleration, *v* is the ego car (EC) speed, $\tilde{v}$ is the EC’s desired speed, β is the acceleration exponent, *s* is the relative distance between EC and front car (FC), $\tilde{s}$ is the desired relative distance as defined in (8), $s_{0}$ is the minimal gap at standstill, *T* is the desired time headway, Δv is the speed difference between EC and FC, and *b* is the comfortable deceleration.

$\dot{a}$ is the desired acceleration of the vehicle. In this equation, the second item in parentheses measures the gap between the speed and desired speed to promote vehicle acceleration, and the third item measures the gap between the actual distance and desired distance to promote vehicle braking. The desired vehicle distance is defined as follows:

(8) $$\tilde{s}=s_{0}+max\left(0,vT+\frac{v\Delta v}{2\sqrt{a_{max}b}}\right)$$

Table 1 is the common parameter setting of IDM.

#### 2.4.2. Driving Strategies

Driving strategies are interaction rules with other road users that use mathematical formulas to express the idea of keeping a safe distance from other vehicles. The responsibility sensitive safety (RSS) model is proposed to ensure absolute security [38]. It is defined as follows:

(9) $$s_{rss}=v\rho +\frac{1}{2}a_{max}\rho^{2}+\frac{{\left(v+\rho a_{max}\right)}^{2}}{2a_{min,brake}}-\frac{v_{FC}^{2}}{2a_{max}},$$

where $s_{rss}$ is the safety distance, *v* is the EC speed, $v_{FC}$ is the FC speed, ρ is the response time, $a_{min,brake}$ is the minimal braking deceleration until stoppage. Table 2 is the parameter setting of RSS.

As a defensive driving strategy, RSS is assumed to accelerate at maximal acceleration during the reaction time of detecting FC braking and to decelerate at minimal braking speed after the reaction. When facing a dangerous situation, defensive driving actively abandons the right of way to avoid conflict.

A negotiated driving strategy disagrees with the FC’s ownership of the right of way by adjusting the safety distance in the car-following state [39]. The new safety gap should be as follows:

(10) $$s_{n}=v\rho +\frac{v^{2}}{2a_{brake}}-\frac{v_{FC}^{2}}{2a_{max}}.$$

The formula removes the unreasonable acceleration term during the reaction and redefines the braking deceleration as follows:

(11) $$a_{brake}=a_{min,brake}+\frac{v}{v_{max}}\left(a_{max}-a_{min,brake}\right)$$

To evaluate these two strategies, we embedded two safety distances into the IDM model by substituting $s_{rss}$ and $s_{n}$ for $\tilde{s}$.

#### 2.4.3. Simulation Scheme

**Figure 4:**
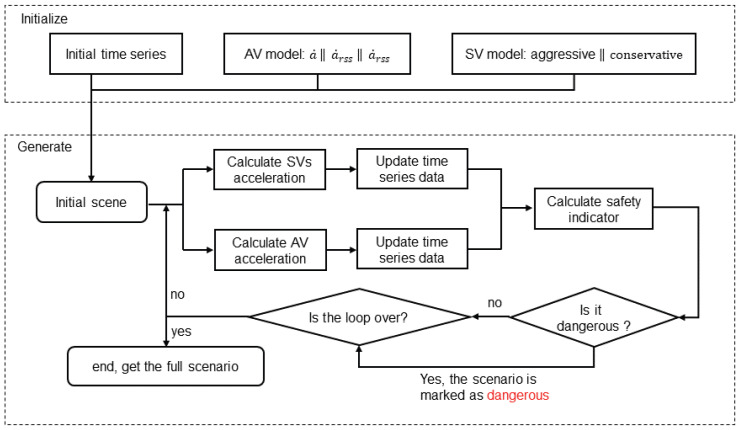
Flowchart of scenario generation.

The goal of scenario generation is to obtain scene sequences through interaction. Calculating the acceleration and updating the timing sequence are repeated until the vehicle passes the test or crashes. The driver model does not sample maneuvers per inference. When the driver decides to change lanes, the maneuver label remains unchanged for 3 s. The inference algorithm for SV is summarized in Algorithm 1. The scenario generation process based on this algorithm is shown in Figure 4.

**Figure 5:**
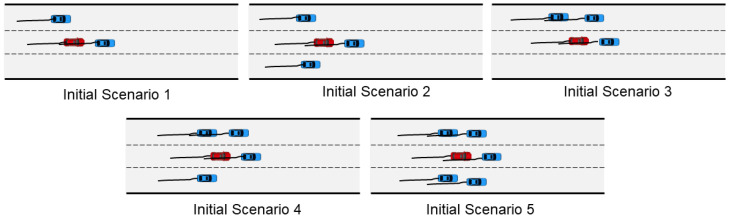
Initialization of five scenarios.

As shown in Figure 5, we first initialized five scenarios of different complexity levels with different vehicle numbers and locations. For each scenario, the basic configuration was to set all SVs as aggressive driver models or all as conservative driver models. Furthermore, the aggressive driver position was set according to the odd–even car number to examine the impact of relative position. In addition, the original driver model was used as a control group for all scenarios. In total, there were 20 experimental groups and 5 control groups. AV controlled by IDM was tested 1000 times in each group, using the time to collision (TTC) as the safety indicator [40]. TTC is defined as the time to collision between the EC and the FC on the current road. The situation is considered dangerous if EC speed is greater than FC speed, and the relative distance is closer.

(12) $$\mathrm{TTC}\left(t\right)=\left\{\begin{array}{cc} \frac{|x_{\mathrm{FC}}\left(t\right)-x_{\mathrm{EC}}\left(t\right)|-L}{v_{\mathrm{EC}}\left(t\right)-v_{\mathrm{FC}}\left(t\right)} & v_{\mathrm{EC}}\left(t\right)>v_{\mathrm{FC}}\left(t\right)\\ \infty & v_{\mathrm{EC}}\left(t\right)\leq v_{\mathrm{FC}}\left(t\right) \end{array}\right.$$

where *L* is the length of the car, $x_{\mathrm{FC}}\left(t\right)$ is the position of FC, $x_{\mathrm{EC}}\left(t\right)$ is the position of EC, $v_{\mathrm{FC}}\left(t\right)$ is the velocity of FC, $v_{\mathrm{FC}}\left(t\right)$ is the velocity of EC.
**Algorithm 1:**Inference algorithm for SV.Initialize: I;T;t=0;i=0;**while** t++<T and no-collision **do**    **if** $m_{lat}=$ lane change and i++ < 15 **then**        Sample maneuver $m_{lon}$        a← output of SV model;        update *I* by *a*;    **end if**    **if** $m_{lat}=$ keep lane **then**        Sample maneuver $\left(m_{lat},m_{lon}\right)$        a← output of SV model;        update *I* by *a*;    **end if****end while**

TTC is aimed at emergency situations where the distance between vehicles is relatively close and where there is a large speed difference, such as the sudden braking of the vehicle in front, which is a dangerous and urgent situation.

To evaluate the driving strategy, we observed the following distance and safety indicator changes during the test in the randomly selected car-following and cut-in scenarios. It was more appropriate to use another safety indicator, time headway (THW), because the safety distance grows [41]. THW is defined as the time difference between EC and FC passing the same place, and it was calculated by dividing the distance between the two vehicles by EC speed.

(13) $$\mathrm{THW}\left(t\right)=\frac{|x_{\mathrm{FC}}\left(t\right)-x_{\mathrm{EC}}\left(t\right)|}{v_{\mathrm{EC}}\left(t\right)}$$

THW mainly alarms when the distance between vehicles is close, and can help drivers in developing a standardized driving habit to maintain a distance between vehicles. We defined it as a dangerous but not urgent situation. We defined the dangerous threshold as TTC less than 5 s and THW as less than 2 s [42,43].

## 3. Results and Analysis

In this section, we discuss and analyze the results of scenario generation with heterogeneous driver models and verify two driving strategies with our scenarios. The verification results prove the validity of our approach.

### 3.1. Implementation of Scenario Generation

Table 3 shows the evaluation results on our models. Negative log likelihood is a metric in endpoint distribution prediction. Our model outperformed the baseline CS-LSTM [28] because of the mean squared error loss at sampling endpoints during training [44]. CS-LSTM uses convolutional social pooling and generates a unimodal output distribution. We additionally report the cross entropy of maneuver probability. A cross entropy of less than 0.05 for classification tasks indicates good performance.

**Figure 6:**
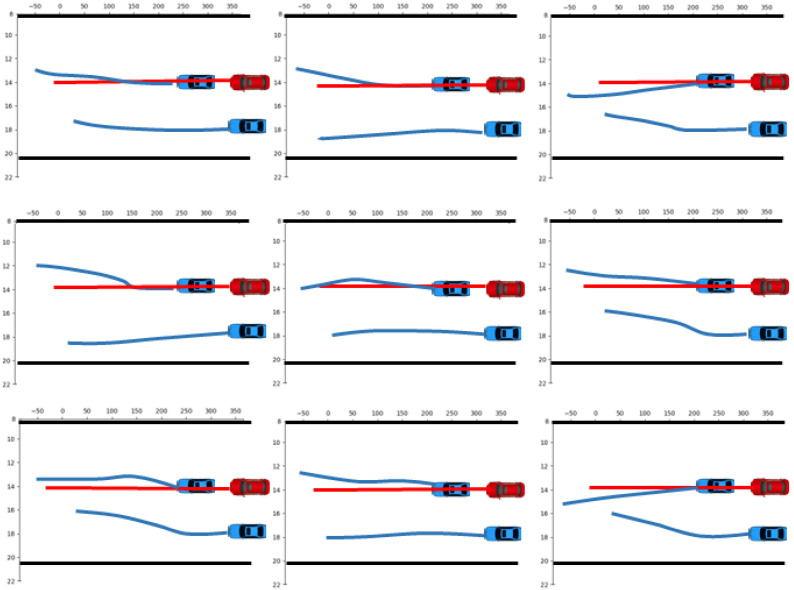
Generated scenario examples.

Figure 6 exemplifies some generated scenarios for a simple situation. The scenarios had smooth curves and could change lanes at any possible moment. If the lane change is not completed due to time constraints, the test time can be extended as needed. Sampling as much as possible enables coverage-oriented test automation. Furthermore, we could achieve accelerated evaluation in two ways. One is the manual control of dangerous maneuvers. For example, it is dangerous to change lanes directly at the beginning, as shown in Figure 6. In Algorithm 1, the initial lateral maneuver could be set as a lane change. The other is to use special sampling methods such as importance sampling methods when sampling endpoints. Combining the two approaches can achieve spatially oriented test automation. Our method is able to generate realistic and plausible scenarios.

According to the experimental design, we tested an AV with the IDM model in 25 groups. Table 4 shows the percentages of challenging scenarios. Compared to the original driver model, the heterogeneous driver models could change the number of challenge scenarios in the scenario space. All SVs set to be aggressive bring more dangerous scenarios. Some situations depict scenarios where conservative vehicles are in front of traffic, also increasing the number of dangerous scenarios. The comparison in the column shows hat the increase in vehicles is also one of the reasons for the increase in dangerous situations.

The results indicate that we could generate more dangerous and complex scenarios by adjusting the location and number of aggressive drivers.

### 3.2. Verification

**Figure 7:**
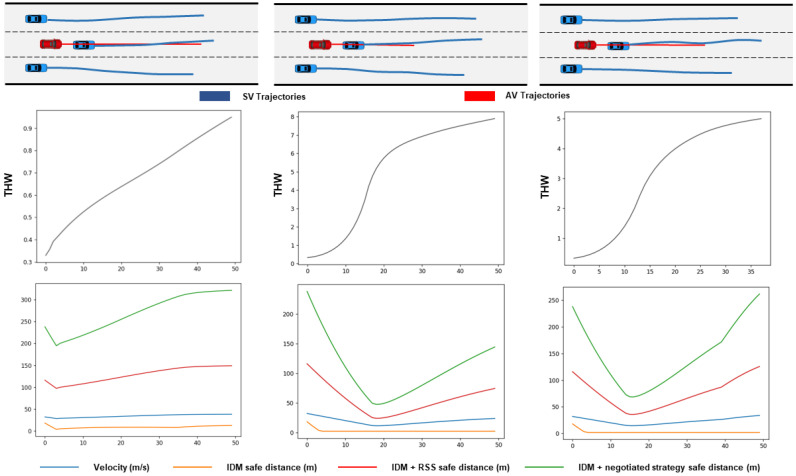
Safety and efficiency evaluation in car-following scenarios.

To demonstrate that the scenarios generated using our driver model are usable, the scenarios are applied to evaluate driving strategies. Given a car-following scenario with an initial THW of less than 2 s, the AV with the original IDM model was continuously dangerous during the test time owing to the close following distance. Figure 7 shows that both driving strategies converge THW from danger to safety in car-following scenarios, but the convergence value of RSS is larger. This is attributable to the more reasonable safety distance of the negotiation strategy.

**Figure 8:**
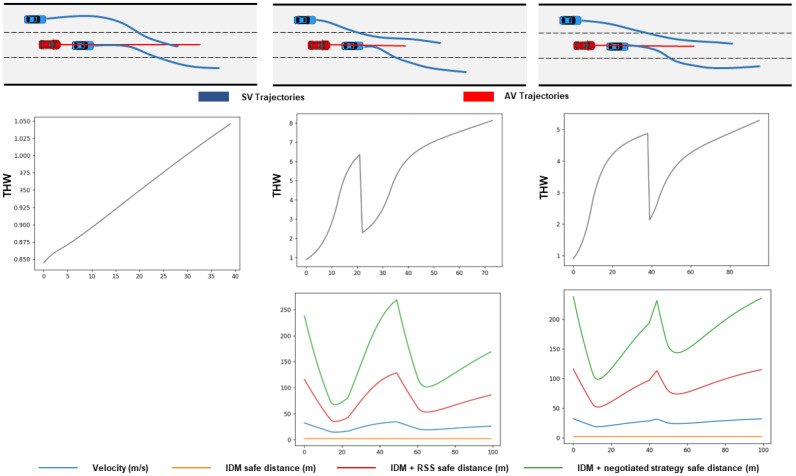
Safety and efficiency evaluation in cut-in scenarios.

As in Figure 8, if a vehicle suddenly cuts in, both strategies could respond in time and brake at a safe distance. The convergence process of THW is similar to that of car following. From the deceleration process, the slope of the speed curve indicates that the negotiation strategy had a shorter deceleration time and smoother braking speed.

Table 5 summarizes the value range of THW and safety distance. RSS can guarantee absolute security, but negotiation policies have higher traffic efficiency. This shows that our verification results are correct and proves the validity of our driver model and method.

## 4. Conclusions

In this paper, we proposed a scenario generation method considering driver heterogeneity. This method improves the number of challenging events in the scenario space by changing the driver model style of the environmental vehicles. Our model quantifies different drivers’ preferences by learning the probability of their behavior. Simulations were implemented in multiple initialization scenarios to demonstrate the role of heterogeneity. The results show that adjusting the number and location of aggressive drivers could lead to more dangerous scenarios and thus improve the efficiency of testing. Thus, the method ensures realism and diversity. Then, we used our scenarios to evaluate conservative strategy and negotiate strategy. The evaluation results show that the conservative strategy was safer and that the negotiation strategy was more efficient, which verified the effectiveness of our approach. The choice of driving strategy depends on the trade-off between safety and efficiency. Cumulatively, our approach could accelerate the testing of AVs. In future work, we could delineate more detailed driver styles or consider heterogeneity from other perspectives. As driver models become more diverse, scenarios become more complex, and danger increases, so our future work could consider these factors.

## Figures and Tables

**Table 1:** Parameters for the IDM model.

Parameter	Description	Value
$\tilde{v}$	Desired speed	40 m/s
β	Acceleration exponent	4
$s_{0}$	Minimal gap	2 m
*T*	Desired time headway	2 s
$a_{max}$	Maximal acceleration	6 m/s${}^{2}$
*b*	Comfortable deceleration	3 m/s${}^{2}$

**Table 2:** Parameters for driving strategies.

Parameter	Description	Value
ρ	Response time	2/3 s
$a_{max}$	Maximal acceleration	6 m/s${}^{2}$
$a_{min,brake}$	Minimal deceleration	3 m/s${}^{2}$

**Table 3:** Evaluation results on our models.

Model	NLL	Cross Entropy
**Aggressive**	2.43	0.018
**Conservative**	2.56	0.025
**Original**	2.17	0.021
**CS-LSTM**	3.30	-

**Table 4:** Percentage of challenging scenarios.

Initial Scenario	All SVs Aggressive	All SVs Conservative	Front SVs Aggressive	Back SVs Aggressive	Original Driver Model
**1**	7%	0%	0%	5%	0%
**2**	1%	0%	0%	5%	0%
**3**	11%	0%	1%	6%	1%
**4**	10%	1%	0%	13%	2%
**5**	9%	1%	0%	6%	2%

**Table 5:** Summary of driving strategies evaluation.

	IDM	IDM + RSS	IDM + Negotiated Strategy
**THW**	0–1 s	0–8 s	0–5 s
**Safety distance**	2–30 m	>50 m	>40 m

## Data Availability

The original contributions presented in the study are included in the article, and further inquiries can be directed to the corresponding author.
